# Increased risk of refeeding syndrome–like hypophosphatemia with high initial amino acid intake in small-for-gestational-age, extremely-low-birthweight infants

**DOI:** 10.1371/journal.pone.0221042

**Published:** 2019-08-23

**Authors:** Se In Sung, Yun Sil Chang, Jin Hwa Choi, Yohan Ho, Jisook Kim, So Yoon Ahn, Won Soon Park

**Affiliations:** Department of Pediatrics, Samsung Medical Center, Sungkyunkwan University School of Medicine, Seoul, Korea; Hopital Robert Debre, FRANCE

## Abstract

**Background:**

Recent nutrition guidelines for extremely-low-birth-weight infants (ELBWIs) recommend implementation of high initial amino acid (AA) supplementation in parenteral nutrition.

**Objective:**

We sought to evaluate the influence of AA intake on refeeding syndrome–like electrolyte disturbances including hypophosphatemia in ELBWIs.

**Study design:**

Medical records of 142 ELBWIs were reviewed. Demographic, nutritional, outcome, and electrolyte data were compared between ELBWIs with initial low (1.5 g/kg/day) and high (3 g/kg/day) AA intake. Multivariate analysis was conducted to determine the odds ratio of hypophosphatemia with high AA intake and small-for-gestational-age (SGA) ELBWIs.

**Results:**

The incidence of hypophosphatemia and severe hypophosphatemia increased from 51% and 8% in period I to 59% and 20% in period II, respectively (p = 0.36 and < 0.01). Specifically, SGA ELBWIs showed higher incidence of hypophosphatemia than appropriate-for-gestational age (AGA) ELBWIs in period II, whereas there was no difference in period I. For severe hypophosphatemia, SGA ELBWIs presented a 27% incidence versus a 2% incidence in AGA ELBWIs, even with low initial AA intake. Despite no difference in phosphate intake between infants with and without hypophosphatemia, serum phosphate level reached a nadir at the sixth postnatal day and gradually recovered over the second week in infants with hypophosphatemia. In multivariate analyses, the odds ratios for severe hypophosphatemia were 3.6 and 6.6 with high AA intake and SGA status, respectively, with the highest being 18.0 with combined high AA intake and SGA status.

**Conclusions:**

In summary, high initial AA intake significantly increased the risk of refeeding syndrome–like electrolyte dysregulations including severe hypophosphatemia in ELBWIs. In SGA ELBWIs, the risk of electrolyte disturbance was significantly higher, even with low initial AA intake. Therefore, new tailored parenteral nutrition protocols starting with lower energy intake and a gradual increase over the first week may be warranted for application in high-risk SGA ELBWIs.

## Introduction

Early nutritional support including higher calories and greater protein intake in the first week of life for extremely-low-birthweight infants (ELBWIs) reduces mortality and the appearance of morbidities such as bronchopulmonary dysplasia and late-onset sepsis and improves neurodevelopmental outcome [1,2]. Recent ESPGHAN/ESPEN/ESPR/CSPEN guidelines on pediatric parenteral nutrition (PN) and Cochrane Database of Systematic Reviews on amino acid intake on PN in newborns recommend implementation of early aggressive nutrition composed of a PN regimen with high initial amino acid (AA) supplementation (2–3 g/kg/day) and initiation of early enteral trophic feeding soon after birth to limit cellular catabolism and promote extrauterine growth [3–7]. However, some clinical data suggest that electrolyte imbalances including hypophosphatemia, hypercalcemia, and hypokalemia might be triggered by early aggressive PN during the first week of life with high AA intake in ELBWIs [8–12]. Therefore, further studies are necessary to determine the safe and efficacious upper limits of initial energy and AA intake necessary to promote postnatal growth without toxicities in ELBWIs.

Refeeding syndrome, characterized by hallmark electrolyte dysregulation of hypophosphatemia along with hypokalemia and hypomagnesemia, is an uncommon but potentially fatal phenomenon that occurs in undernourished patients receiving aggressive nutritional rehabilitation [13,14]. However, the incidence and risk factors of refeeding syndrome–like electrolyte dysregulation in ELBWIs have not been elucidated. Several studies have reported that early aggressive PN with high initial AA intake in micropreemies increased the risk of early refeeding syndrome–like electrolyte disturbances such as hypophosphatemia and hypokalemia [15–18]. Additionally, small for gestational age (SGA) micropreemies suffering from intrauterine growth restriction due to chronic undernourishment during the fetal period have been reported to develop refeeding syndrome–like severe hypophosphatemia and hypokalemia following initiation of PN soon after birth [10,11,15,16]. Beginning in October 2013, we changed the PN protocol from low (1.5 g/kg/day) to high (3 g/kg/day) initial AA intake in ELBWIs. In the present retrospective observational study, we reviewed the medical records of ELBWIs with a 23- to 28-week gestational age to determine whether our PN protocol change from low to high initial AA intake was associated with improved or worsened adverse refeeding syndrome–like hypophosphatemia, especially in SGA ELBWIs.

## Materials and methods

Data collection was approved by the Institutional Review Board (IRB) of Samsung Medical Center (SMC) in Seoul, Korea, which allowed for a waiver of informed consent requirements for this retrospective chart review (IRB no. SMC 2016-09-112).

### Study population

The medical records of 142 ELBWIs with birth weight less than 1,000 g and gestational age between 23 and 28 weeks who were admitted to the SMC Neonatal Intensive Care Unit (NICU) from October 2012 to December 2014 were reviewed retrospectively. We arbitrarily divided the study into Period I (October 2012 to September 2013; n = 55) and Period II (October 2013 to December 2014; n = 87) according to the PN protocol of our NICU with low and high initial AA intake, respectively.

### Definition of variables

The lower limit of the reference range of serum phosphate level is higher in premature infants (5 mg/dL, 1.6 mmol/L) than in adults (3 mg/dL, 1.0 mmol/L) according to the ESPGHAN/ESPEN/ESPR/CSPEN guidelines [3,4]. Therefore, we defined hypophosphatemia as one or more episodes of serum phosphate level being less than 5 mg/dL. As mild hypophosphatemia (2.5–5 mg/dL) is common in ELBWIs owing to the suboptimal phosphate supply during the first one or two weeks of life regardless of SGA status or AA intake, we also investigated the incidence of severe hypophosphatemia defined as one or more episodes of serum phosphate level being less than 2.5 mg/dL during the first two weeks of life. In addition, the following cutoffs were used to define electrolyte imbalance: hypercalcemia (serum calcium 10 > mg/dL), hypokalemia (serum potassium < 3.0 mg/dL), and hypomagnesemia (serum magnesium < 1.5 mg/dL). Demographic data comprised gestational age, birthweight, SGA status, antenatal steroid, and preeclampsia/eclampsia. Gestational age was determined by maternal last menstrual period and the modified Ballard test. SGA was defined as birthweight less than the 10th percentile according to the intrauterine growth curve reported in 2010 [19].

Neonatal outcome data included mortality, intraventricular hemorrhage (grades III–IV), retinopathy of prematurity requiring laser therapy, patent ductus arteriosus, bronchopulmonary dysplasia, necrotizing enterocolitis, and blood culture–proven sepsis. The definition of bronchopulmonary dysplasia used was oxygen or positive-pressure ventilator dependency at a postmenstrual age of 36 weeks. Data of duration of invasive and noninvasive mechanical ventilation, insulin treatment, nosocomial infection, mortality during the first 2 weeks, and early postnatal growth data were also collected.

For nutritional data, those on enteral feeding, energy intake of parenteral and enteral nutrition, and nutrient (glucose, AA, lipid) intake were collected. Calcium and phosphate intake within the first two weeks of life was also recorded. The data on the intake amount that we collected were based on the administered daily intake, rather than the prescribed amounts. The deficit of phosphate intake was obtained by subtracting the actual phosphate intake from the estimated phosphate requirement. The estimated phosphate requirement was calculated based on the following formula: P requirement = Ca intake/2.15 + (AA intake -1.3)*0.8*12.3 [7], where, phosphate requirement and calcium intake were expressed in mg/kg/day and AA intake was expressed in g/kg/day.

Blood urea nitrogen (BUN) level was additionally noted as a measure of nutritional status.

### Nutrition protocol

The nutrition protocol of our NICU for ELBWIs was as follows: (1) a central line was placed just after birth, and PN was initiated as soon as possible within the first 24 hours; (2) minimal enteral feeding with human breast milk or preterm milk of 10 to 20 mL/kg/day was initiated within the first hours of life; and (3) enteral feeding of 20 to 30 mL/kg/day was usually maintained during the first two weeks and increased to full enteral feeding with respiratory and hemodynamic stability.

The composition of PN on Day 1 during Period I was typically dextrose of 7.5 g/kg/day, AA of 1.5 g/kg/day, and lipid of 1 g/kg/day. Then, AA administration was gradually increased to maintain a nonprotein calorie-to-AA ratio of 30 to 40:1. Prompted by a literature review [2,18], the PN protocol during Period II was changed to include high AA (3.0–3.5 g/kg/day) within the first 24 hours of life from the previous low initial AA (1.5 g/kg/day) administration during Period I. The amount of AA intake was subsequently adjusted according to BUN level and urine output in both periods. Calcium gluconate and potassium phosphate were administrated at a molar ratio of 1:1 to 1.3:1. The summation of calcium (mEq/L) plus phosphate (mM/L) was usually limited to 40 to 60 due to the concern of crystal formation. We monitored the serum ionized calcium level and adjusted calcium and phosphate intake on a daily basis.

### Blood sampling protocol

Blood samples to test chemistry and electrolyte profiles were obtained via umbilical arterial blood in the delivery room or from the infant’s arterial or venous blood within the first 24 hours after admission to NICU. Subsequent sampling was usually done during the first 48 to 72 hours of life to test serum electrolyte levels. Then, the following sampling was done regularly with an interval of five to seven days or urgently if necessary. Sodium, potassium, and ionized calcium levels were obtained daily via a bedside point-of-care-test (i-STAT Analyzer; Abbott Point Of Care Inc., Princeton, NJ, USA).

### Incidence and risk factors of hypophosphatemia

**Fig 1:**
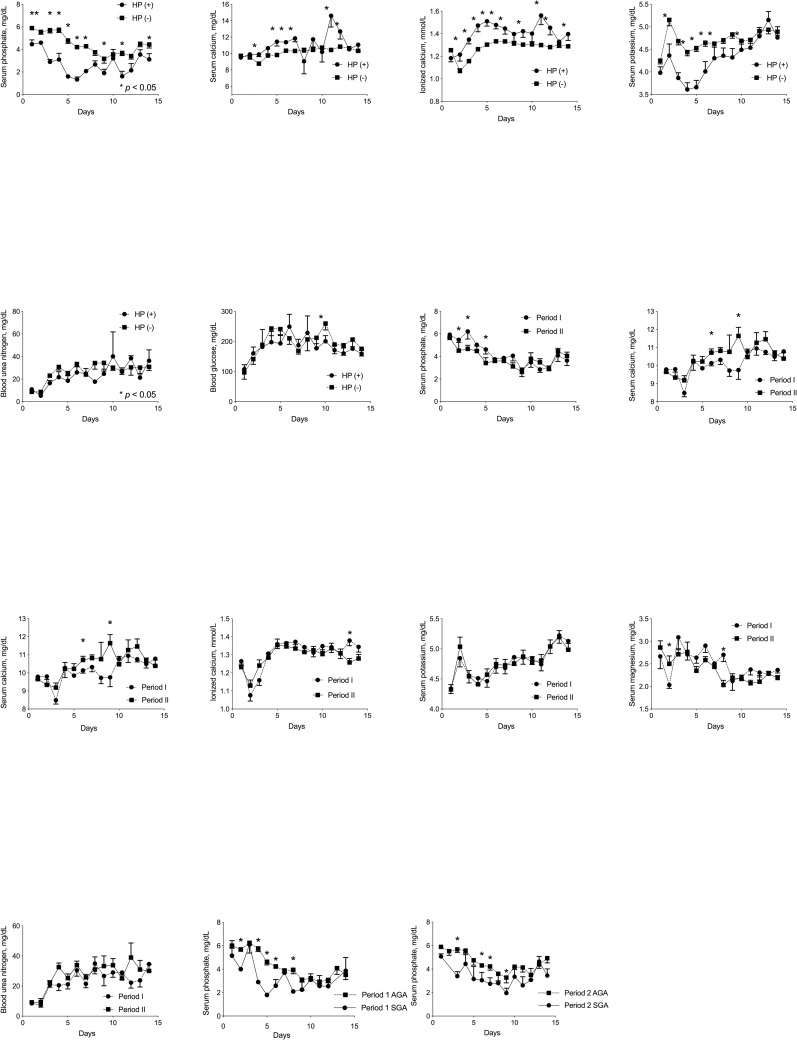
Changes in serum phosphate, total serum calcium, ionized calcium, potassium, magnesium, and BUN levels during the first two weeks of life.

The incidences of hypophosphatemia and severe hypophosphatemia were compared between infants born during Periods I and II (Table 1). Due to the theoretical risk of hypophosphatemia, especially in SGA ELBWIs, infants were subdivided according to SGA status. Fig 1 illustrates temporal changes in serum phosphate, calcium, potassium, magnesium, glucose, and BUN levels in ELBWIs with and without hypophosphatemia during the first two postnatal weeks of life. To evaluate the risk factors of hypophosphatemia, clinical characteristics were also compared between infants with and without hypophosphatemia (Table 2). The intake of macronutrients and calcium/phosphate was compared between Periods I and II and in the hypophosphatemia (+) and (−) groups in each period (Table 3). To investigate the effects of high initial AA intake and SGA status on hypophosphatemia, adjusted odds ratios (aORs) with 95% confidence intervals (CIs) were calculated.

**Table 1:** Incidence of hypophosphatemia in Period I and II according to SGA status.

	Total (n = 142)			
	Period I (n = 55)		Period II (n = 87)	
	SGA (n = 11)	AGA (n = 44)	SGA (n = 26)	AGA (n = 61)
**Hypophosphatemia, n (%)**	8 (73)	20 (45)	21 (81)*	30 (49)
**Severe hypophosphatemia, n (%)**	3 (27)*	1 (2)	14 (54)*	3 (5)
***Demographic characteristics***				
**Gestational age, weeks**	26.7 ± 2.4*	24.8 ± 1.5	27.0 ± 2.7*	25.2 ± 1.5
**Birth weight, g**	571 ± 151*	739 ± 125	618 ± 190*	783 ± 132
**Male, n (%)**	7 (64)	24 (55)	13 (50)	32 (53)
**Cesarean section, n (%)**	10 (90)	34 (77)	26 (100)*	45 (74)
**Antenatal steroid, n (%)**	8 (73)	37 (84)	22 (85)	53 (87)
**Preeclampsia/eclampsia**	5 (45)*	1 (2)	6 (23)*	1 (2)
**Apgar score (1 min)**	4.2 ± 1.4	4.3 ± 1.3	4.4 ± 1.4	4.7 ± 1.3
**Apgar score (5 min)**	6.0 ± 1.3	6.7 ± 1.4	6.8 ± 1.3	7.2 ± 1.3
***Neonatal outcomes***				
**Mortality, n (%)**	4 (36)	6 (14)	4 (15)	3 (5)
**Intraventricular hemorrhage (grade 3, 4), n (%)**	2 (18)	8 (18)	1 (4)	4 (7)
**Retinopathy of prematurity requiring laser, n (%) therapy, n (%)**	1 (9)	11 (25)	3 (12)	9 (16)
**Bronchopulmonary dysplasia, n (%)**	2 (18)	20 (45)	9 (35)	19 (31)
**Necrotizing enterocolitis (stage 2, 3), n (%)**	4 (36)	6 (14)	4 (15)	9 (15)
**Blood culture positive sepsis, n (%)**	3 (27)	14 (32)	5 (19)	12 (20)
***Clinical disorders related to hypophosphatemia***				
**Duration of mechanical ventilation, days**	39.6 ± 24.9	37.3 ± 22.2	32.4 ± 27.3	31.0 ± 36.4
**Duration of noninvasive respiratory support, days**	25.3 ± 19.7	28.8 ± 21.9	19.5 ± 21.9*	31.8 ± 23.5
**Insulin therapy during the first 2 weeks, n (%)**	4 (36)	16 (36)	10 (38)	24 (39)
**Nosocomial infections during the first 2 weeks, n (%)**	2 (18)	5 (11)	2 (8)	10 (16)
**Mortality during the first 2 weeks, n (%)**	1 (9)	1 (2)	1 (4)	1 (2)
***Early postnatal growth data during the first 2 weeks***				
**Days needed to regain birth weight**	6.6 ± 5.2*	11.7 ± 5.1	7.3 ± 5.1*	12.9 ± 6.0
**Weight gain, g/kg/day**	12.8 ± 6.8	16.2 ± 4.8	15.7 ± 6.0	18.8 ± 4.0
**Maximal weight loss, %**	6.3 ± 2.9*	11.7 ± 4.9	5.7 ± 6.7	9.1 ± 7.5

SGA, small for gestational age; AGA, appropriate for gestational age

* P < 0.05 compared with AGA infants within the same period

**Table 2:** Demographic and outcomes data of the study population.

	Period I (n = 55)		Period II (n = 87)		Total (n = 142)	
	HP (+) (n = 28)	HP (−) (n = 27)	HP (+) (n = 51)	HP (−) (n = 36)	HP (+) (n = 78)	HP (−) (n = 64)
***Hypophosphatemia*, *n (%)***	*28/55 (51)*		*51/87 (59)*		*79/142 (56)*	
***Severe hypophosphatemia*, *n (%)***	*4/55 (8)*		*17/87 (20)*		*21/121 (17)*	
***Demographic characteristics***						
**Gestational age, weeks**	25.4 ± 2.1	24.9 ± 1.6	25.9 ± 2.0	25.5 ± 2.2	25.7 ± 2.1	25.3 ± 2.0
**Birthweight, g**	704 ± 140	708 ± 154	695 ± 167	787 ± 158	699 ± 162*	752 ± 156
**Small for gestational age, n (%)**	8 (29)	3 (11)	21 (41)*	5 (14)	28 (36) *	9 (14)
**Male, n (%)**	17 (61)	14 (52)	31 (61)	14 (39)	48 (62)	28 (44)
**Cesarean section, n (%)**	21 (75)	23 (85)	46 (90)	25 (69)	67 (86)	48 (75)
**Antenatal steroid, n (%)**	23 (82)	22 (82)	41 (80)	34 (94)	64 (82)	56 (88)
**Preeclampsia/eclampsia**	5 (18)	1 (4)	5 (10)	2 (4)	10 (13)	3 (5)
**Apgar score (1 min)**	4.4 ± 1.4	4.0 ± 1.3	4.5 ± 1.4	4.9 ± 1.3	4.5 ± 1.4	4.5 ± 1.3
**Apgar score (5 min)**	6.9 ± 1.4	6.3 ± 1.5	7.1 ± 1.3	7.2 ± 1.3	7.0 ± 1.3	6.8 ± 1.5
***Neonatal outcome***						
**Mortality during hospitalization, n (%)**	6 (21)	4 (15)	3 (6)	4 (11)	9 (12)	8 (13)
**Intraventricular hemorrhage (grades 3 and 4), n (%)**	7 (25)	3 (11)	2 (4)	3 (8)	9 (12)	6 (9)
**Retinopathy of prematurity requiring laser therapy, n (%)**	4 (14)	8 (30)	4 (8)	8 (22)	8 (11)	16 (27)
**Bronchopulmonary dysplasia, n (%)**	11 (39)	11 (41)	19 (37)	9 (25)	30 (38)	20 (31)
**Necrotizing enterocolitis (stages 2 and 3), n (%)**	6 (21)	4 (15)	7 (14)	6 (17)	13 (17)	10 (16)
**Blood culture (+) sepsis, n (%)**	8 (29)	9 (33)	9 (18)	8 (22)	17 (22)	17 (27)
***Clinical disorders related to hypophosphatemia***						
**Duration of mechanical ventilation, days**	40 ± 25	37 ± 22	38 ± 38*	23 ± 24	39 ± 34	29 ± 24
**Duration of noninvasive respiratory support, days**	25 ± 20	29 ± 22	27 ± 23	29 ± 24	27 ± 22	29 ± 23
**Insulin therapy during the first 2 weeks, n (%)**	9 (32)	11 (41)	24 (47)	10 (28)	33 (42)	21 (33)
**Nosocomial infections during the first 2 weeks, n (%)**	3 (11)	4 (15)	6 (12)	6 (17)	9 (12)	10 (16)
**Mortality during the first 2 weeks, n (%)**	1 (4)	1 (4)	0 (0)	2 (6)	1 (1)	3 (5)
***Early postnatal growth data during the first 2 weeks***						
**Days needed to regain birth weight**	10.0 ± 6.3	11.3 ± 4.6	10.4 ± 6.5	12.6 ± 5.9	10.3 ± 6.4	12.0 ± 5.3
**Weight gain, g/kg/day**	15.3 ± 5.7	15.7 ± 5.1	17.5 ± 3.7	18.4 ± 6.2	16.7 ± 4.6	17.2 ± 5.9
**Maximal weight loss, %**	10.1 ± 5.4	11.3 ± 4.6	7.9 ± 6.8	8.3 ± 8.2	9.6 ± 7.0	8.6 ± 6.4

HP, hypophosphatemia

* P < 0.05 compared with HP (**−**) within the same period

**Table 3:** Comparison of the nutritional characteristics between Periods I and II.

	Total (n = 142)			
	Period I (n = 55)		Period II (n = 87)	
	HP (+) (n = 28)	HP (−) (n = 27)	HP (+) (n = 51)	HP (−) (n = 36)
***Parenteral nutrition***				
***AA intake*, *g/kg/day***				
**Day 1,**	1.6 ± 0.3	1.6 ± 0.3	2.7 ± 0.6*	2.7 ± 0.7*
**Day 3**	2.1 ± 0.4	1.9 ± 0.4	3.0 ± 0.3*	3.0 ± 0.3*
**Day 7**	2.6 ± 0.5	2.5 ± 0.5	2.8 ± 0.6	3.0 ± 0.5
**Day 10**	2.5 ± 0.4	2.5 ± 0.5	2.8 ± 0.6	2.9 ± 0.5
**Day 14**	2.5 ± 0.4	2.4 ± 0.4	2.5 ± 0.5	2.8 ± 0.6
***Glucose intake*, *g/kg/day***				
**Day 1**	7.6 ± 0.4	7.5 ± 0.4	7.8 ± 0.4	7.7 ± 0.3
**Day 3**	9.0 ± 0.9	9.1 ± 0.9	9.7 ± 1.0	9.7 ± 0.9
**Day 7**	11.3 ± 1.8	11.6 ± 1.4	12.3 ± 1.6	12.8 ± 1.5
**Day 10**	12.2 ± 1.4	12.3 ± 1.3	13.2 ± 1.5	13.4 ± 1.4
**Day 14**	13.0 ± 1.2	12.7 ± 1.7	13.8 ± 1.3	14.0 ± 1.1
***Lipid intake*, *g/kg/day***				
**Day 1**	0.7 ± 0.2	0.7 ± 0.2	0.8 ± 0.2	0.9 ± 0.4
**Day 3**	1.2 ± 0.4	1.0 ± 0.4	1.4 ± 0.4	1.3 ± 0.4
**Day 7**	1.8 ± 0.6	1.7 ± 0.5	2.0 ± 0.6	1.9 ± 0.6
**Day 10**	1.9 ± 0.67	2.0 ± 0.7	2.2 ± 0.6	2.2 ± 0.6
**Day 14**	2.1 ± 0.6	2.0 ± 0.7	2.3 ± 0.7	2.2 ± 0.9
***Calcium intake*, *mEq/kg/day***				
**Day 1**	1.2 ± 0.1	1.2 ± 0.1	1.3 ± 0.5	1.6 ± 0.6
**Day 3**	1.3 ± 0.6	1.6 ± 0.6	2.0 ± 0.9	2.7 ± 0.7
**Day 7**	1.2 ± 0.9	1.4 ± 0.8	1.4 ± 1.1	2.6 ± 1.0
***Phosphate intake*, *mM/kg/day***				
**Day 1**	0.4 ± 0.2	0.3 ± 0.2	0.4 ± 0.3	0.7 ± 0.4
**Day 3**	0.6 ± 0.2	0.6 ± 0.2	0.9 ± 0.3	1.2 ± 0.3
**Day 7**	0.8 ± 0.4	0.6 ± 0.2	1.0 ± 0.4	1.2 ± 0.4
***Deficit of phosphate intake (estimated need–actual intake)*, *mM/kg/day***				
**Day 1**	0.5 ± 0.2	0.6 ± 0.2	0.8 ± 0.3	0.8 ± 0.4
**Day 3**	0.6 ± 0.5	0.7 ± 0.4	1.0 ± 0.3	0.9 ± 0.3
**Day 7**	0.4 ± 0.7	0.7 ± 0.4	0.5 ± 0.8	1.0 ± 0.6
**Cumulative deficit (day 1–5)**	2.5 ± 1.2	2.7 ± 1.3	3.7 ± 1.5*	4.2 ± 1.0*
***Energy intake (parenteral)*, *kcal/kg/day***				
**Day 1**	40.0 ± 3.7	40.0 ± 1.7	51.9 ± 3.5	52.5 ± 5.5
**Day 7**	67.7 ± 10.8	68.0 ± 8.1	72.0 ± 8.2	75.4 ± 9.8
***Enteral nutrition*, *mL/kg/day***				
**Day 1**	5 ± 4	5 ± 4	5 ± 4	9 ± 9
**Day 7**	24 ± 10	20 ± 8	25 ± 14	31 ± 23

HP, hypophosphatemia; AA, amino acid

* P < 0.05 compared with Period I within the same hypophosphatemia group

### Statistical analysis

Statistical differences between SGA and appropriate-for-gestational-age (AGA) infants were calculated using the Chi square test for categorical variables and a t-test or Mann–Whitney U test for quantitative variables. Binary logistic regression was used for multivariable analysis, testing the effects of early high AA, SGA status, and the combination of early high AA and SGA status on hypophosphatemia. Gestational age was adjusted for each multivariable analysis. A p-value less than 0.05 was considered statistically significant. Statistical analysis was executed using STATA version 13.1 (StataCorp LP, College Station, TX, USA).

## Results

### Changes in incidence of hypophosphatemia and electrolyte abnormalities

The incidence of hypophosphatemia increased from 51% in period I to 59% in period II, although the change was not significant (p = 0.36) (Tables 1 and 2). The incidence of hypophosphatemia was higher in SGA infants than in AGA infants (78% versus 48%, p < 0.01). Specifically, SGA ELBWIs showed higher incidence of hypophosphatemia than AGA ELBWIs in period II (81% versus 49%, p = 0.01), whereas there was no difference between SGA and AGA infants in period I. However, for severe hypophosphatemia (< 2.5 mg/dL), the incidence of 7% (4/55) during period I significantly increased to 20% (17/87) during period II (p = 0.04). The SGA ELBWIs presented a 27% incidence of severe hypophosphatemia versus a 2% in AGA ELBWIs, even with low initial AA intake during period I (p = 0.01).

Fig 1 illustrates temporal changes in serum phosphate, calcium, potassium, magnesium, glucose, and BUN levels in ELBWIs according to the presence of hypophosphatemia, the periods, and the SGA status. While there were no significant differences in blood glucose and BUN levels or significantly higher serum calcium and significantly lower serum potassium and magnesium levels, serum phosphate level in the hypophosphatemia (+) group reached a nadir at six days after birth and was significantly lower than that in the hypophosphatemia (−) group throughout the first two postnatal weeks.

### Changes in demographic and outcomes data

While the rate of SGA was significantly higher and birthweights were significantly lower in the hypophosphatemia (+) group versus the hypophosphatemia (−) group, there were no significant differences in other demographic data including gestational age among the study periods and groups (Table 2).

In period 2, the duration of invasive mechanical ventilation was longer in hypophosphatemia (+) than hypophosphatemia (-). Otherwise, there were no significant differences in outcomes variables including mortality between study periods and groups (Table 2).

### Changes in nutrient intake

Table 3 presents a comparison of macronutrients and calcium/phosphate intake in PN according to period of hypophosphatemia (+) and (−). The AA intake from Days 1 to 3 during Period II was significantly higher than that during Period I (1.6 ± 0.3 vs. 2.8 ± 0.5; p = 0.03) but was not significantly different after Day 7. There was no difference in phosphate intake between hypophosphatemia (+) and (-). Other variables including glucose and caloric intake were not significantly different between study periods and groups. The cumulative deficit of phosphate intake during the first 5 days before the nadir of hypophosphatemia was higher in period II than in period I (2.6 ± 1.3 versus 4.0 ± 1.3 mM/kg/day, p < 0.01).

### Odds ratio of hypophosphatemia

In multivariate analysis, early high AA intake did not increase the risk of hypophosphatemia (< 5 mg/dL) with OR of 1.2 (95% CI: 0.6–2.4). However, SGA and the combination of early high AA intake and SGA increased the risk of hypophosphatemia, with ORs of 3.4 (95% CI: 1.5–8.0) and 3.5 (1.2–10.2), respectively. For severe hypophosphatemia (< 2.5 mg/dL), early high AA intake, SGA, and the combination of early high AA intake and SGA increased the risk, with ORs of 3.6 (95% CI: 1.3–10.4), 6.6 (95% CI: 3.4–12.9), and 18.0 (95% CI: 6.0–53.8), respectively.

## Discussion

Interruption of continuous placental nutritional flow after birth increases the risk of nutrient deficiency in ELBWIs. The recent ESPGHAN/ESPEN/ESPR/CSPEN guidelines on pediatric PN and also the recent Cochrane Database Systematic Reviews on amino acid supplementation on PN in newborns recommended initial amino acid supplementation with high AA (3g/kg/day) and energy intake [3,4]. The recent introduction of an early aggressive PN in ELBWIs has been shown to prevent early inadequate nutrition–induced metabolic disturbances such as hyperphosphatemia, hypocalcemia, nonoliguric hyperkalemia, and hypoglycemia by inhibiting cellular catabolism and promoting growth [9,20,21]. However, emerging evidence showed that early aggressive PN also facilitated refeeding syndrome–like electrolyte disturbances such as hypophosphatemia, hypercalcemia, hypokalemia, and hypomagnesemia, especially in SGA ELBWIs, at the end of first postnatal week.[9,10] Moreover, the incidence and extent of hypophosphatemia showed a significant association with AA intake amount in these infants [9–11]. In the present study, the difference of the incidence of hypophosphatemia (< 5 mg/dL) between period I and II did not reach statistical significance. This result might be probably due to the high incidence of mild hypophosphatemia in ELBWIs resulted from the cumulative deficit of postnatal phosphate supply. However, the incidence of severe hypophosphatemia (< 2.5 mg/dL) was significantly increased in period II compared with that in period I. The high amino acid intake, followed by transient hyperinsulinemia and accelerated cellular anabolism, increased the requirement of phosphate and other intracellular ions. Our results showed that the deficit of phosphate intake (estimated requirement–actual intake) during the first 5 days before the nadir of hypophosphatemia was higher in period II than in period I (2.6 ± 1.3 versus 4.0 ± 1.3 mM/kg/day, p < 0.01). Because there was no difference in the phosphate intake between the two periods, the increased cumulative deficit in period II might be a result of an increased estimated requirement of phosphate due to augmented AA anabolism. The lower phosphate supply since birth, combined with a scarce deposition of minerals in the bone and higher protein anabolism led to early hypophosphatemia, which was severe in the undernourished SGA infants.

However, the optimal amounts for AA and phosphate intake in high-risk infants remain unclear. Bustos LG et al. reported that an intermediate dose of AA (2.0–2.5 mg/kg/day) decreased the incidence of early hypophosphatemia in these patients [22]. In our study, even the intermediate-risk infants, SGA infants in period I and AGA infants in period II, showed a significant incidence of early-onset severe hypophosphatemia. The optimal doses of AA should be set individually between low and intermediate doses according to the severity of preexisting undernourishment.

In the previous studies by Mulla S et al. and Bustos LG et al., the authors postulated that the conventional Ca-to-P molar ratio of 1.3:1 might be insufficient to meet the phosphate requirement in infants with preexisting phosphate depletion [22,23]. Taken together, the low to intermediate dose of AA at the initiation of PN along with high phosphate supply at the Ca-to-P molar ratio of 1:1 or less might be necessary for SGA ELBWIs. The gradual increase in AA intake according to a nonprotein calorie-to-AA ratio of 30 to 40:1 up to the end of the first postnatal week should be accompanied by an increase in phosphate intake based on daily phosphate requirement. Further randomized clinical trials are necessary to validate this practice.

For the mechanism of refeeding syndrome–like electrolyte disturbances, abrupt introduction of high exogenous AA stimulates endogenous insulin secretion followed by intracellular shifts of phosphate, potassium, and magnesium ions in the SGA ELBWIs with depleted body stores of these ions [9,24]. As depletion of serum phosphate level would lead to failure of adenosine triphosphate (ATP) generation, infants with severe hypophosphatemia are at a higher risk of respiratory muscle fatigue resulting in respiratory failure [18]. Moreover, as depletion of ATP following severe hypophosphatemia can also lead to phagocyte dysfunction [12], infants with severe hypophosphatemia might have an increased risk of sepsis. In this study, hypophosphatemia that reached nadir at Postnatal Day 6 was transient, and infants recovered in the second week of life. As most ELBWIs regardless of hypophosphatemia were ventilator-dependent during the first two weeks of life, no significant differences in mortality or onset of morbidities including BPD according to presence of transient hypophosphatemia were observed in this study. However, in period II, the duration of invasive mechanical ventilation was prolonged in infants with hypophosphatemia compared with those without hypophosphatemia. More research is needed to confirm whether this result is due to refeeding syndrome-associated hypophosphatemia. On the other hand, there were no difference in the incidence of sepsis or nosocomial infection between infants with and without hypophosphatemia.

Limitations of this study include its retrospective observational study design and performance in a single tertiary center NICU. Nutritional management was individualized; therefore, the type of milk and the amounts of AA, calcium, and phosphate intake in PN were not controlled strictly in this study. As SGA infants were arbitrarily defined as those falling under less than the 10th percentile of birthweight, the pathologic etiology due to a chronically undernourished intrauterine environment caused by various maternal or placental factors such as preeclampsia, diabetes, or cigarette smoking could not be discriminated from constitutional causes of SGA in this study.

## Conclusions

In the present study, high initial AA intake of 3 g/k/g/day increased the risk of refeeding syndrome-like electrolyte dysregulation in SGA ELBWIs. Moreover, the risk of severe hypophosphatemia (< 2.5mg/dL) was significantly higher even with a low initial AA intake of 1.5 g/kg/day in SGA (< 10th percentile) ELBWIs. A new tailored PN protocol starting with lower to intermediate AA (1.5–2.0 g/kg/day) and higher phosphate supply (Ca to P molar ratio of 1:1 or less), and gradually increasing the AA and phosphate intake over the first postnatal week might be necessary for high-risk SGA ELBWIs.

## Supporting information

S1 File Anonymized dataset. (XLSX)

## Abbreviations

EPI: extremely preterm infant
AA: amino acid
PN: parenteral nutrition
